# State-Dependent Alterations of Hippocampal Theta–Gamma Coupling in 5xFAD Mice and Their Differential Modulation by Donepezil

**DOI:** 10.3390/brainsci16080816

**Published:** 2026-07-31

**Authors:** Haodong Wang, Yajun Wang, Zhengyang Lv, Yueting Deng, Chao Pan, Yuping Li, Zikai Zhou

**Affiliations:** 1 Zhongshan Institute for Drug Discovery, Shanghai Institute of Materia Medica, Chinese Academy of Sciences, Zhongshan 528400, China; wanghaodong1147@zidd.ac.cn (H.W.); zhengyang_lv@163.com (Z.L.); dengyueting1243@zidd.ac.cn (Y.D.); panchao0465@zidd.ac.cn (C.P.); liyuping@zidd.ac.cn (Y.L.); 2 School of Basic Medicine and Clinical Pharmacy, China Pharmaceutical University, Nanjing 211198, China; 3 School of New Chinese Materia Medica, Nanjing University of Chinese Medicine, Nanjing 210023, China; wangyajun956@zidd.ac.cn; 4 School of Pharmacy, Zunyi Medical University, Zunyi 563000, China; 5 School of Pharmacy, University of Chinese Academy of Sciences, Beijing 101408, China

**Keywords:** Alzheimer’s disease, 5xFAD, theta–gamma coupling, phase–amplitude coupling, donepezil, hippocampus

## Abstract

**Highlights:**

**What are the main findings?**

- 5xFAD mice show reduced hippocampal theta–low gamma coupling and elevated theta–high gamma coupling.
- Donepezil preferentially enhances theta–low gamma coupling, with limited normalization of theta–high gamma coupling.

**What are the implications of the main findings?**

- Theta–low gamma coupling may serve as a treatment-responsive readout of cholinergic modulation in AD models.
- Persistent theta–high gamma elevation may reflect a stable AD-related hippocampal network abnormality.

**Abstract:**

Background/Objectives: Alzheimer’s disease (AD) is associated with progressive hippocampal circuit dysfunction, but electrophysiological measures of state-dependent abnormalities and treatment responsiveness remain incompletely characterized. We examined hippocampal theta–gamma coupling in 5xFAD mice across behavioral states and after donepezil. Methods: Local field potentials were recorded from CA1 in freely behaving wild-type (WT) and 5xFAD mice during home-cage activity, open-field exploration, and Y-maze testing. Power spectral density and theta–gamma phase–amplitude coupling (PAC) were quantified at the animal level at baseline and after seven days of donepezil. Results: Untreated 5xFAD mice showed reduced theta–low-gamma coupling during home-cage and open-field activity, but not Y-maze exploration, and elevated theta–high-gamma coupling in all three contexts. Donepezil increased theta–low-gamma coupling during open-field and Y-maze exploration and produced a partial numerical shift toward WT levels in the home cage. For theta–high-gamma coupling, the treated group did not differ significantly from either comparator. Untreated 5xFAD mice also showed reduced center exploration and distance traveled in the open field, while Y-maze spontaneous alternation was unchanged. Theta–high-gamma coupling correlated positively with open-field center time in WT mice only. Conclusions: Hippocampal theta–gamma coupling shows frequency- and context-dependent abnormalities in 5xFAD mice. Theta–low-gamma coupling is sensitive to short-term cholinergic modulation during exploration, whereas donepezil’s effect on theta–high-gamma coupling remains inconclusive. Animal-level PAC may provide a candidate functional readout, but longitudinal and cross-model validation is required before biomarker claims are justified.

## 1. Introduction

Alzheimer’s disease (AD) is the most common cause of dementia and is clinically characterized by progressive cognitive decline, with major pathological features including amyloid-β (Aβ) deposition, tau pathology, synaptic dysfunction or loss, neuroinflammation, and neurodegeneration [1,2,3,4]. Although these molecular and cellular abnormalities are central to AD pathogenesis, they do not fully explain how pathological changes lead to impaired information processing and cognitive decline. Increasing evidence indicates that neuronal hyperactivity, excitation–inhibition imbalance, altered oscillatory activity, and disrupted circuit organization can emerge early, sometimes before extensive neurodegeneration or overt cognitive symptoms become evident [5,6,7,8]. These findings suggest that network-level dysfunction is not merely a downstream consequence of neuronal loss, but an active component of AD progression. Preclinical evaluation of AD models and therapeutic interventions has traditionally relied on neuropathological and behavioral assessments. Measurements of amyloid burden, tau-related pathology, gliosis, synaptic integrity, neuronal loss, and neuroinflammatory responses provide important information about disease pathology, whereas behavioral paradigms such as the Morris water maze, Y-maze, and novel object recognition tests are widely used to evaluate learning and memory performance in AD mouse models [9,10,11,12]. However, behavioral outcomes can be influenced by non-cognitive factors, including locomotor activity, anxiety-like behavior, sensory or visual function, motivation, and variations in testing protocols or environmental conditions [9,13]. In contrast, conventional endpoint histological measurements provide spatially resolved information about pathological changes but offer limited insight into ongoing neuronal dynamics. Therefore, electrophysiological measures that objectively and quantitatively assess circuit dysfunction may provide complementary candidate functional measures for characterizing disease-associated circuit dysfunction and treatment responsiveness [14].

Local field potentials (LFPs) are extracellular voltage fluctuations that reflect the spatial and temporal summation of transmembrane currents in neuronal populations near the recording electrode, with substantial contributions from synchronized postsynaptic activity. Intrinsic membrane currents, action-potential-related afterpotentials, and volume-conducted sources may also contribute [15,16,17]. Thus, the term ‘local’ does not imply that an LFP originates exclusively from neurons immediately adjacent to the electrode; its effective spatial reach depends on source geometry, neuronal synchrony, signal frequency, tissue conductivity, and the recording and reference configurations [18]. LFPs should therefore be interpreted as composite population-level signals rather than direct measurements of individual-neuron firing. Because they capture rhythmic activity across a broad frequency range, LFPs are well suited for evaluating neural oscillations and cross-frequency interactions associated with hippocampal network function.

In the hippocampus, theta- and gamma-range LFP oscillations are strongly implicated in behavioral-state regulation, information processing, and memory-related functions [19,20,21]. Interactions between theta and gamma rhythms have become an important framework for understanding hippocampal circuit function. Phase–amplitude coupling (PAC) is a widely studied form of cross-frequency interaction in which the phase of a slower oscillation modulates the amplitude of a faster oscillation. This mechanism is thought to coordinate neuronal activity across different temporal scales and to organize information processing within distributed networks [22,23,24]. In the hippocampus, theta–gamma coupling has been implicated in episodic memory encoding and retrieval, spatial representation, and working-memory-related processing [25,26]. Experimental and computational studies further suggest that theta rhythms provide a temporal framework within which gamma activity can organize hippocampal information flow [27]. Accordingly, alterations in theta–gamma coupling may provide complementary functional indicators of circuit dysfunction beyond conventional behavioral or endpoint pathological measurements.

Importantly, theta–gamma coupling is not a single uniform process. Distinct gamma frequency ranges may reflect partially different hippocampal inputs and functional operations. Low-gamma activity is commonly associated with CA3–CA1 communication, whereas high-gamma activity has often been linked to entorhinal cortex–CA1 input [21,28]. This suggests that theta–low-gamma and theta–high-gamma coupling may represent dissociable modes of hippocampal information processing [29,30,31]. Separating these coupling components may therefore provide more specific information about AD-related network abnormalities than analyzing broadband gamma activity alone.

Abnormal neuronal oscillations and cross-frequency interactions have increasingly been reported in both patients with AD and experimental AD models. Importantly, PAC abnormalities in AD are heterogeneous rather than uniformly reduced, and their direction and functional significance appear to depend on the brain region, frequency pair, behavioral state, and stage of disease. Studies in patients with AD have identified altered low-frequency phase–gamma amplitude coupling in medial temporal and parahippocampal regions, with coupling patterns further influenced by the presence of subclinical epileptiform activity and network hyperexcitability [32,33]. In animal models, reductions in hippocampal theta–gamma coupling have been observed during early or pre-plaque stages, whereas increased or spatially redistributed PAC has also been reported in cortical networks under specific behavioral conditions. These apparently divergent findings suggest that different forms of PAC may reflect distinct pathophysiological processes, ranging from impaired mnemonic coordination within hippocampal circuits to compensatory synchronization or pathological network hyperexcitability.

Recent studies further illustrate the frequency and task specificity of AD-related PAC alterations. In APP/PS1 mice performing a spatial working-memory task, theta–low-gamma coupling within and between the ventral hippocampus and medial prefrontal cortex was reduced during memory encoding, and the reduction was associated with impaired behavioral performance [34]. Because that study focused on theta–low-gamma coupling and directed information flow, it does not establish whether theta–high-gamma coupling was preserved or altered. Complementing these gamma-range findings, a recent wideband intracranial EEG study in 5xFAD mice identified increased high-frequency oscillation activity and altered coupling between slow-wave phase and HFO amplitudes at a young, pre-pathological stage before the appearance of overt amyloid plaques or neuroinflammatory changes [35]. Because HFO-related PAC is generally associated more closely with abnormal population synchronization and network hyperexcitability, it may represent a pathophysiologically distinct phenomenon from theta–gamma coupling involved in hippocampal information processing. Together, these observations demonstrate that AD can affect multiple forms of PAC, including theta–low-gamma, theta–high-gamma, and slow-wave–HFO coupling, each potentially capturing different aspects of circuit dysfunction.

The 5xFAD mouse model was selected to address the specific question of how amyloid-associated pathology affects hippocampal network coordination across behavioral states. This model overexpresses human APP and PSEN1 carrying five familial AD-associated mutations and consequently develops rapid and robust Aβ42 accumulation, amyloid plaque deposition, gliosis, synaptic abnormalities, and alterations in neuronal network activity [36,37,38,39,40]. At approximately 4 months of age—the stage examined in the present study—amyloid pathology is evident in the hippocampus and cortex, and early abnormalities in hippocampal theta- and gamma-range activity have been reported [37,40]. These features provide a reproducible experimental window in which amyloid-associated circuit dysfunction can be examined before the most advanced stages of neurodegeneration. Nevertheless, 5xFAD mice do not reproduce the full clinicopathological spectrum of human AD. In particular, they do not develop progressive human-like tau aggregation or neurofibrillary tangles, and their pathology is driven by neuronal overexpression of familial APP and PSEN1 mutations, whereas most human AD cases are sporadic, multifactorial, and progress over decades [36,37]. The model should therefore be regarded as an accelerated amyloid-dominant model rather than a comprehensive model of sporadic AD. Its relevance to the present study lies in enabling controlled investigation of the relationship between Aβ-associated network dysfunction, behavioral context, and cholinergic modulation.

Electrophysiological biomarkers may also be useful for assessing pharmacological modulation of AD-related circuit dysfunction. Donepezil is an acetylcholinesterase inhibitor used for symptomatic AD treatment, increasing acetylcholine availability at cholinergic synapses [41]. Cholinergic signaling contributes to hippocampal theta generation, attention, learning, and memory, and cholinergic manipulation can modulate gamma oscillations and theta–gamma coupling [42,43,44]. These findings raise the possibility that PAC-derived measures may provide sensitive readouts of cholinergic engagement at the circuit level. However, whether theta–low-gamma and theta–high-gamma coupling exhibit similar or distinct responsiveness to donepezil treatment in AD models remains insufficiently defined.

In this study, we recorded local field potentials from the hippocampal CA1 region of freely behaving WT and 5xFAD mice during home-cage activity, open-field exploration, and Y-maze testing. Power spectral density and theta–gamma phase–amplitude coupling were quantified, with separate analyses of theta–low-gamma and theta–high-gamma coupling. We further assessed the effects of seven consecutive days of donepezil treatment on these electrophysiological measures. We hypothesized that 5xFAD mice would exhibit frequency-specific and behavioral-state-dependent alterations in hippocampal theta–gamma coupling and that cholinergic enhancement would differentially modulate low- and high-gamma-associated PAC components. By combining electrophysiological recordings with behavioral assessments across multiple awake contexts, this study sought to characterize amyloid-associated hippocampal circuit dysfunction and to evaluate PAC-derived measures as candidate functional indicators of treatment responsiveness. In contrast to previous studies focusing on interregional coupling during a single memory phase or on cortical slow-wave–HFO interactions, our study specifically examined local CA1 theta–gamma PAC across distinct behavioral states and its modulation by pharmacological cholinergic enhancement.

## 2. Materials and Methods

### 2.1. Animals

Adult male C57BL/6 wild-type (WT) mice and 5xFAD transgenic mice (4 months old, 22–25 g) were used. The age of 4 months was selected as an early stage at which amyloid-associated synaptic and network dysfunction is established but severe behavioral deterioration may not yet dominate task performance. Only male mice were included to reduce sex-related variability in this initial electrophysiological cohort; this choice limits generalizability and requires replication in sex-balanced cohorts. Animals were purchased from Shanghai Model Organisms Center, Inc., Shanghai, China and housed under specific pathogen-free conditions with ad libitum access to food and water on a 12 h light/dark cycle (07:00–19:00) at 22–24 °C and 50–60% humidity. Following electrode implantation, mice were singly housed until electrophysiological recordings were completed. Behavioral tests and recording order were randomized. Behavioral scoring and electrophysiological preprocessing and quantification were performed using coded animal identifiers by investigators blinded to genotype and treatment group; group codes were disclosed only after the predefined outcomes had been extracted. All procedures were approved by the Institutional Animal Care and Use Committee of the Zhongshan Institute for Drug Discovery, Chinese Academy of Sciences, and were conducted in accordance with institutional and national guidelines. The analyzed cohort comprised 7 WT mice and 8 5xFAD mice; the same 8 5xFAD mice contributed post-donepezil measurements where stated. Analysis-specific exclusions are reported in the figure legends.

### 2.2. Genotyping of 5xFAD Mice

5xFAD mice were maintained on a C57BL/6J genetic background by breeding hemizygous transgenic males with C57BL/6J WT females. This breeding strategy generated 5xFAD transgenic mice and WT littermate controls. The 5xFAD line carries human amyloid precursor protein (APP) and presenilin 1 (PSEN1) transgenes harboring five familial AD-associated mutations: APP Swedish (K670N/M671L), APP Florida (I716V), APP London (V717I), PSEN1 M146L, and PSEN1 L286V [36]. Genotyping was performed by polymerase chain reaction (PCR) using genomic DNA extracted from tail biopsies. The primer sequences used for genotyping are provided in Supplementary Table S1. Offspring carrying the 5xFAD transgene were assigned to the AD model group, whereas PCR-negative WT littermates were used as controls. Only genotype-confirmed animals were included in subsequent electrophysiological and behavioral experiments.

### 2.3. Electrode Implantation

Mice were anesthetized with isoflurane (RWD Life Science Co., Ltd., Shenzhen, China) and positioned in a stereotaxic apparatus (RWD Life Science Co., Ltd., Shenzhen, China) for electrode implantation. Eight-channel microwire array electrodes (nickel–titanium alloy; 25 μm wire diameter; 300 μm inter-electrode spacing; Suzhou Kedou Brain Computer Technology Co., Ltd., Suzhou, China) were implanted into the hippocampal CA1 region using the following coordinates relative to bregma: anteroposterior (AP), −2.00 mm; mediolateral (ML), +1.80 mm; dorsoventral (DV), −1.40 mm. Stainless-steel skull screws (RWD Life Science Co., Ltd., Shenzhen, China) were used to stabilize the implant and served as ground and reference electrodes. The electrode assembly was secured with dental acrylic (Shanghai New Century Dental Materials Co., Ltd., Shanghai, China).

Electrophysiological recordings were initiated after a postoperative recovery period of 5–7 days, when mice had recovered from surgery and showed normal spontaneous activity. Following completion of the recordings, electrode placements were verified histologically. Animals with incorrect electrode placement or poor signal quality were excluded from subsequent analyses.

### 2.4. Experimental Design and Donepezil Treatment

After postoperative recovery, baseline home-cage, open-field, and Y-maze recordings were obtained from WT and 5xFAD mice. Donepezil (Shanghai Aladdin Biochemical Technology Co., Ltd., Shanghai, China; 1 mg/kg, intraperitoneally) was then administered to the 5xFAD mice once daily for seven consecutive days. The dose was selected by conversion from the clinically used dose to a mouse-equivalent dose and was within the range used in preclinical rodent studies [41,44]; it should not be interpreted as direct clinical equivalence. Post-treatment electrophysiological and behavioral recordings began on day 8, the day after the seventh daily dose, and used the same procedures and the same 5xFAD animals, enabling within-subject evaluation of treatment-associated changes. No time-matched vehicle-treated 5xFAD group or WT-plus-donepezil group was included. Hippocampal acetylcholine levels and acetylcholinesterase activity were not measured, so cholinergic target engagement was not directly verified in this cohort.

### 2.5. LFP Acquisition and Preprocessing

Local field potentials (LFPs) were recorded from freely moving mice using a multichannel data-acquisition system (Plexon Inc., Dallas, TX, USA). Signals were sampled at 1 kHz and band-pass filtered online between 0.5 and 250 Hz. Recordings were visually inspected offline using coded animal identifiers. Segments containing prominent movement artifacts, excessive noise, unstable baseline drift, or sharp transients were excluded before spectral and PAC estimation. Channels with persistent contamination or histologically confirmed incorrect electrode placement were excluded according to predefined quality-control criteria.

Only high-quality channels located within hippocampal CA1 were retained. Electrophysiological measures were calculated separately for each valid channel and then averaged across valid channels within each mouse to obtain one animal-level value for every recording condition. Thus, channels were treated as technical subsamples, and the mouse was the independent biological replicate.

### 2.6. Power Spectral Density and Phase–Amplitude Coupling Analysis

Data were analyzed using NeuroExplorer 5 (64-bit; Nex Technologies, Colorado Springs, CO, USA) and MATLAB R2023a (MathWorks, Natick, MA, USA). Power spectral density (PSD) was estimated with Welch’s averaged modified periodogram method using a 2048-sample window, 50% overlap, and a 4096-point fast Fourier transform over 0–100 Hz. For quantitative summaries, band power was averaged for each mouse after eligible channels had been averaged within the animal. Visual differences in spectra were not interpreted as effects unless supported by the corresponding animal-level statistical comparison.

Phase–amplitude coupling (PAC) was analyzed in MATLAB using functions from the Buzcode toolbox (master branch, GPL-3.0; Buzsáki Laboratory, GitHub repository; accessed on 14 February 2024). PAC was quantified with the modulation index (MI), which measures the extent to which the amplitude of a higher-frequency oscillation is modulated by the phase of a lower-frequency rhythm [23]. The PAC regions of interest were defined a priori as theta phase (6–9 Hz) coupled to low-gamma amplitude (30–50 Hz) or high-gamma amplitude (65–100 Hz). These rectangular ranges were based on rodent hippocampal literature and the study hypothesis rather than peaks in individual comodulograms. A sensitivity analysis using modestly shifted adjacent band boundaries yielded the same principal directional pattern; this check was treated as supportive rather than independent validation. PAC was estimated from artifact-screened data, but no phase-shuffling, permutation, or surrogate distribution was used to threshold the comodulograms. Consequently, residual coupling related to non-sinusoidal waveforms, broadband transients, sharp-wave or epileptiform events, and filtering cannot be excluded. Home-cage recordings were not segmented into sleep and wake epochs, and open-field and Y-maze analyses were not matched across groups for movement speed, immobility, or exploratory epochs.

For each behavioral condition, MI was first calculated separately for every eligible CA1 channel. Channel-level estimates were then averaged within mouse, and the resulting single animal-level value was used for group comparisons and PAC–behavior correlations. The animal, rather than the recording channel, was the unit of inference. Analysis-specific animal numbers are reported in the corresponding figure legends.

### 2.7. Statistical Analysis

Statistical analyses were performed using GraphPad Prism 10.1.2. Behavioral, PSD, and PAC measures were analyzed at the animal level, with each mouse treated as one independent biological replicate. Bars show the mean ± SEM; box plots show the median and interquartile range with individual animal-level values overlaid. Differences among WT, untreated 5xFAD, and donepezil-treated 5xFAD groups were assessed using one-way analysis of variance followed by Tukey’s multiple-comparisons test. Associations between PAC measures and behavioral outcomes were assessed using simple linear regression from one PAC value per animal, with R^2^ and exact *p* values reported. Given the small cohort and potential influence of locomotion and outliers, correlation analyses were considered exploratory; no multivariable adjustment was performed because the sample was insufficient for stable estimation. Statistical significance was set at *p* < 0.05, and analysis-specific *n* values are reported in the figure legends.

## 3. Results

### 3.1. Experimental Design and Representative LFP Recordings

**Figure 1:**
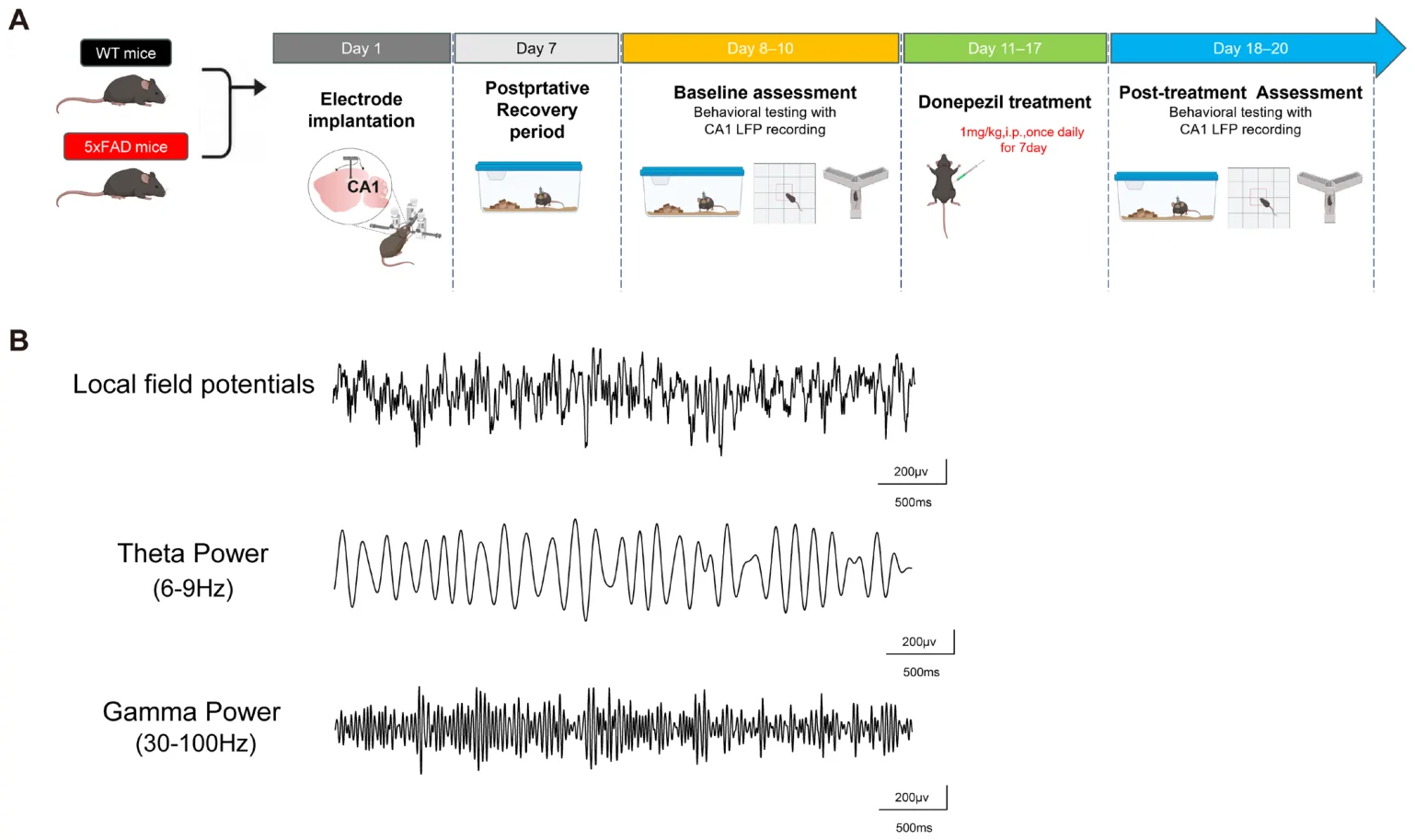
Experimental design and representative hippocampal CA1 local field potential signals. (**A**) Experimental timeline for wild-type (WT) and 5xFAD mice. CA1 electrodes were implanted on day 1, followed by postoperative recovery on days 2–7 and baseline behavioral testing with simultaneous CA1 local field potential (LFP) recording on days 8–10. Donepezil-treated 5xFAD mice received donepezil (1 mg/kg, intraperitoneally) once daily on days 11–17; post-treatment behavioral testing with CA1 LFP recording began on treatment day 8 (study day 18) and was performed on study days 18–20. (**B**) Representative raw CA1 LFP trace and band-pass-filtered theta (6–9 Hz) and gamma (30–100 Hz) traces. Scale bars, 500 ms and 200 µV.

To assess hippocampal network activity in freely behaving mice, microelectrodes were implanted into the CA1 region, and local field potentials (LFPs) were recorded from WT and 5xFAD mice. The experimental timeline is shown in Figure 1A. After electrode implantation and recovery, mice underwent baseline home-cage recording, open-field testing (OFT), and Y-maze testing. Donepezil was then administered during the intervention period, followed by post-treatment OFT and Y-maze assessments.

Representative CA1 LFP traces are shown in Figure 1B. The raw LFP signal was further filtered into theta-band activity and gamma-range activity for subsequent spectral and phase–amplitude coupling analyses. Electrode placement in the CA1 region was verified histologically. This experimental design enabled longitudinal assessment of hippocampal oscillatory activity and behavioral performance before and after cholinergic modulation.

### 3.2. Home-Cage Hippocampal Theta–Gamma Coupling Is Bidirectionally Altered in 5xFAD Mice

**Figure 2:**
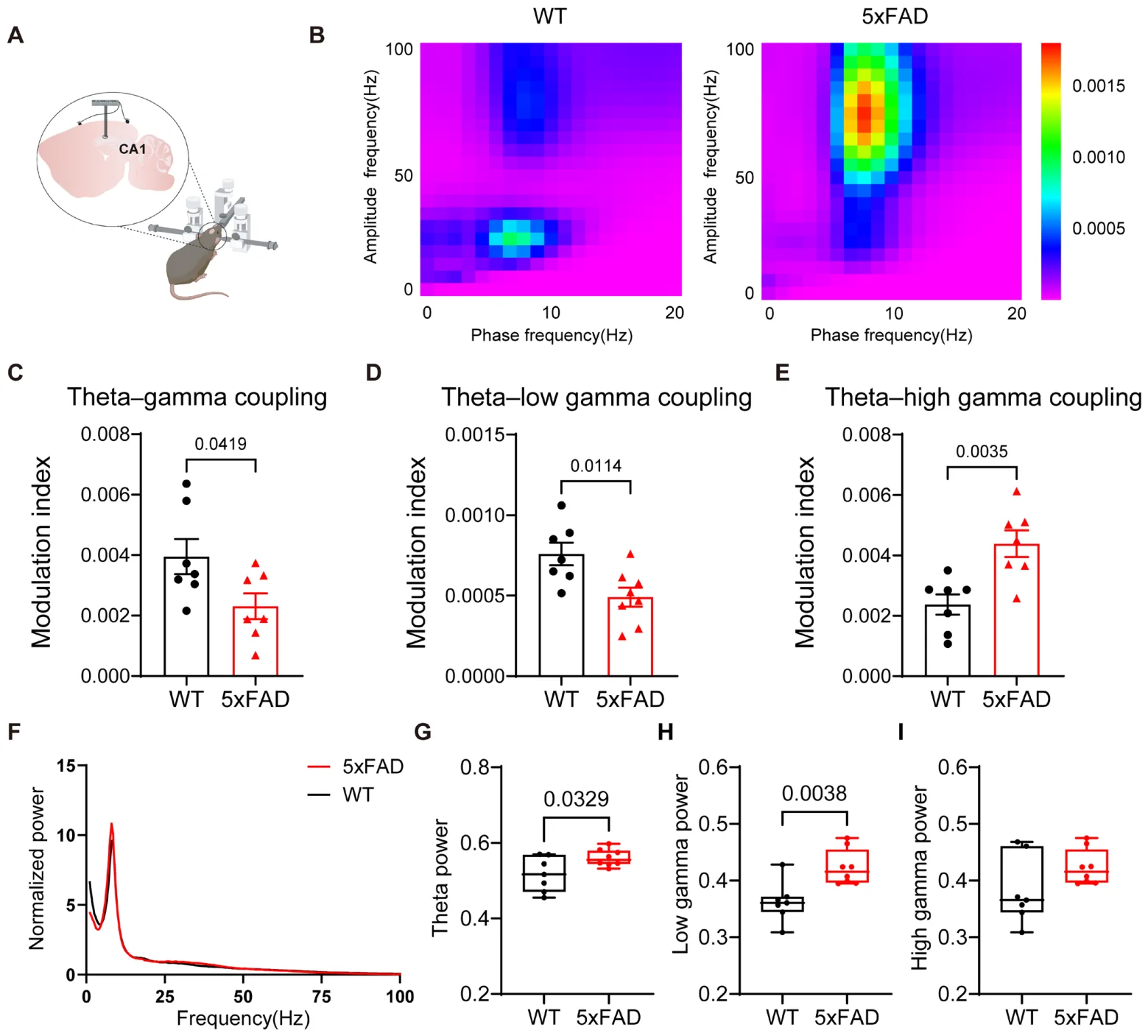
Home-cage CA1 theta–gamma coupling and spectral power in untreated 5xFAD mice. (**A**) Schematic of electrode implantation in the hippocampal CA1 region. (**B**) Representative phase–amplitude coupling (PAC) comodulograms from WT and 5xFAD mice. (**C**–**E**) Modulation index (MI) for overall theta–gamma, theta–low-gamma (6–9 Hz phase and 30–50 Hz amplitude), and theta–high-gamma coupling (6–9 Hz phase and 65–100 Hz amplitude), respectively. (**F**) Normalized power spectral density over 0–100 Hz. (**G**–**I**) Theta-band (6–9 Hz), low-gamma-band (30–50 Hz), and high-gamma-band (65–100 Hz) power, respectively. Bars in (**C**–**E**) show the mean ± SEM; box plots in (**G**–**I**) show the median and interquartile range, with individual values overlaid. Black circles and red triangles denote WT and 5xFAD mice, respectively. Each symbol represents one mouse (animal-level value): WT, *n* = 7; 5xFAD, *n* = 8. Group comparisons were performed using one-way analysis of variance followed by Tukey’s multiple-comparisons test. Exact *p* values for the indicated comparisons were: (**C**) *p* = 0.0419; (**D**) *p* = 0.0114; (**E**) *p* = 0.0035; (**G**) *p* = 0.0329; and (**H**) *p* = 0.0038. No significant group difference was detected in (**I**).

We first analyzed CA1 LFPs recorded under home-cage conditions to evaluate spontaneous hippocampal network activity in untreated WT and 5xFAD mice (Figure 2A). Representative phase–amplitude coupling (PAC) comodulograms revealed distinct theta–gamma coupling patterns between groups (Figure 2B). Animal-level analysis showed that overall theta–gamma PAC was lower in 5xFAD mice than in WT controls (Figure 2C; *p* = 0.0419). Frequency-specific analysis further showed reduced theta–low-gamma coupling (Figure 2D; *p* = 0.0114) and increased theta–high-gamma coupling (Figure 2E; *p* = 0.0035) in 5xFAD mice.

Normalized power spectral density (PSD) was then examined across 0–100 Hz (Figure 2F). Although the overall spectral profiles were broadly similar, animal-level band summaries showed higher theta power (6–9 Hz; Figure 2G; *p* = 0.0329) and low-gamma power (30–50 Hz; Figure 2H; *p* = 0.0038) in 5xFAD mice than in WT mice. High-gamma power (65–100 Hz; Figure 2I) did not differ significantly between groups.

Together, these findings indicate that untreated 5xFAD mice exhibit frequency-specific disruption of home-cage CA1 theta–gamma synchronization, characterized by reduced theta–low-gamma coupling and increased theta–high-gamma coupling. These PAC alterations were accompanied by higher theta- and low-gamma-band power, whereas high-gamma-band power was unchanged.

### 3.3. Donepezil Modulates Home-Cage CA1 Theta–Gamma Coupling in 5xFAD Mice

**Figure 3:**
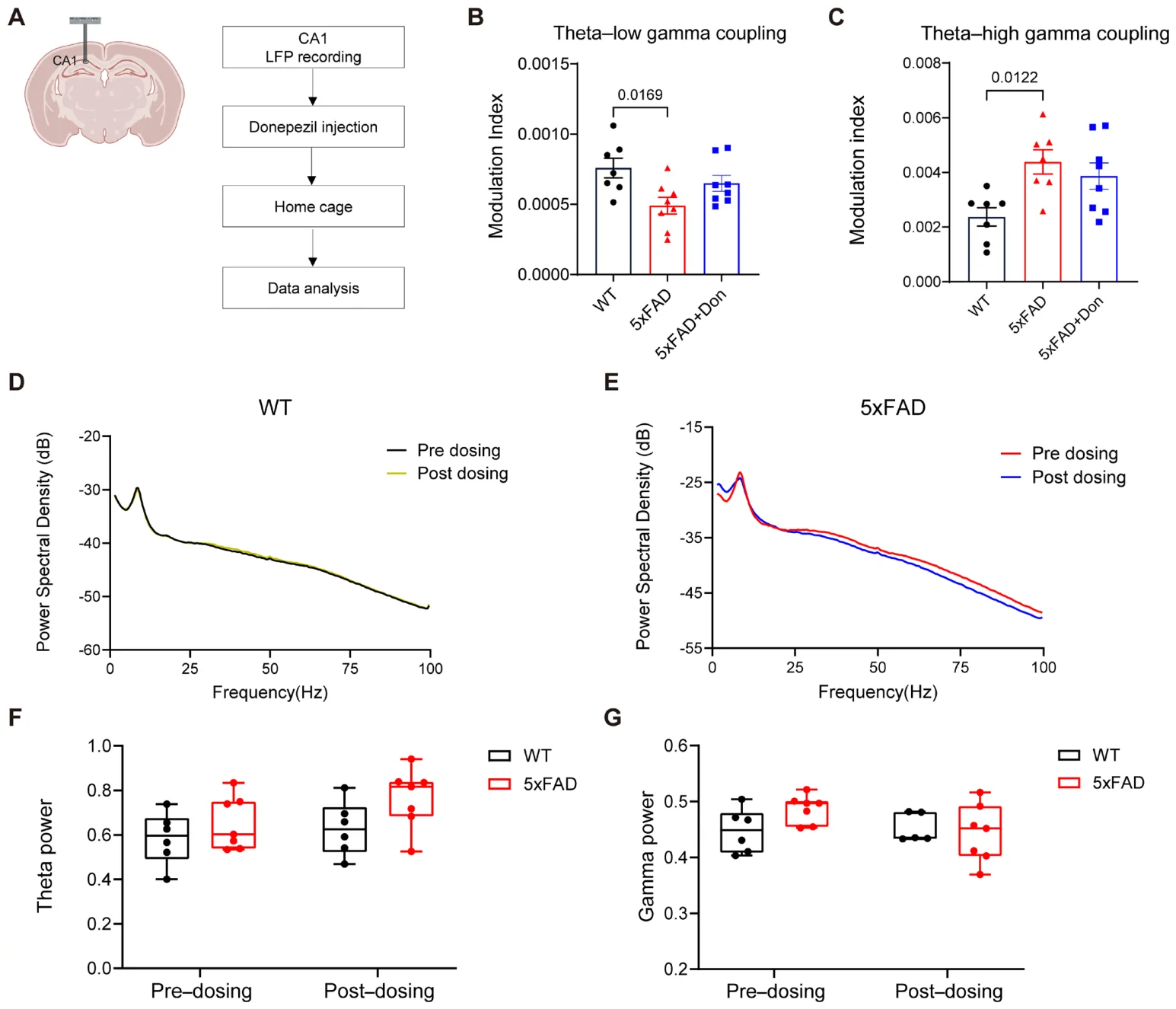
Effects of donepezil on home-cage CA1 theta–gamma coupling and spectral power in 5xFAD mice. (**A**) Experimental workflow for CA1 LFP recording, donepezil administration, home-cage recording beginning on treatment day 8, and data analysis. (**B**,**C**) Theta–low-gamma and theta–high-gamma MI, respectively, in WT, untreated 5xFAD, and donepezil-treated 5xFAD (5xFAD + Don) mice. (**D**,**E**) Power spectral density curves before and after the dosing interval in WT and 5xFAD mice, respectively. (**F**,**G**) Theta- and gamma-band power before and after the dosing interval. Bars in (**B**,**C**) show the mean ± SEM; box plots in (**F**,**G**) show the median and interquartile range, with individual values overlaid. Black circles, red triangles, and blue squares denote WT, untreated 5xFAD, and 5xFAD + Don mice, respectively. Each symbol represents one mouse (animal-level value): WT, *n* = 7; 5xFAD, *n* = 8; 5xFAD + Don, *n* = 8. Group comparisons in (**B**,**C**) were performed using one-way analysis of variance followed by Tukey’s multiple-comparisons test. Exact *p* values for WT versus untreated 5xFAD mice were (**B**) *p* = 0.0169 and (**C**) *p* = 0.0122.

To determine whether cholinergic modulation alters hippocampal network abnormalities in 5xFAD mice, CA1 LFPs were recorded under home-cage conditions in WT, untreated 5xFAD, and donepezil-treated 5xFAD mice (Figure 3A). Theta–low-gamma PAC was lower in untreated 5xFAD mice than in WT mice (Figure 3B; *p* = 0.0169). The mean value in donepezil-treated 5xFAD mice shifted numerically toward the WT level, but no significant pairwise comparison involving the treated group was indicated.

Theta–high-gamma coupling showed the opposite disease-associated pattern. Untreated 5xFAD mice exhibited higher theta–high-gamma coupling than WT mice (Figure 3C; *p* = 0.0122). The mean value in donepezil-treated 5xFAD mice was intermediate between the WT and untreated 5xFAD groups, but no significant pairwise comparison involving the treated group was indicated.

Power spectral density (PSD) analysis showed minimal changes across the dosing interval in WT mice and modest spectral differences in 5xFAD mice (Figure 3D,E). Quantification of theta- and gamma-band power showed no significant pre- versus post-dosing differences in either genotype (Figure 3F,G). These results indicate that any donepezil-associated change in home-cage PAC was not accompanied by a detectable change in overall theta- or gamma-band power.

Together, these findings confirm opposite disease-associated changes in theta–low-gamma and theta–high-gamma coupling under home-cage conditions. The treated group showed partial numerical shifts toward WT values, but the present comparisons do not establish significant normalization of either PAC component after donepezil treatment.

### 3.4. Donepezil Alters Open-Field Behavior and CA1 Theta–Gamma Coupling in 5xFAD Mice

**Figure 4:**
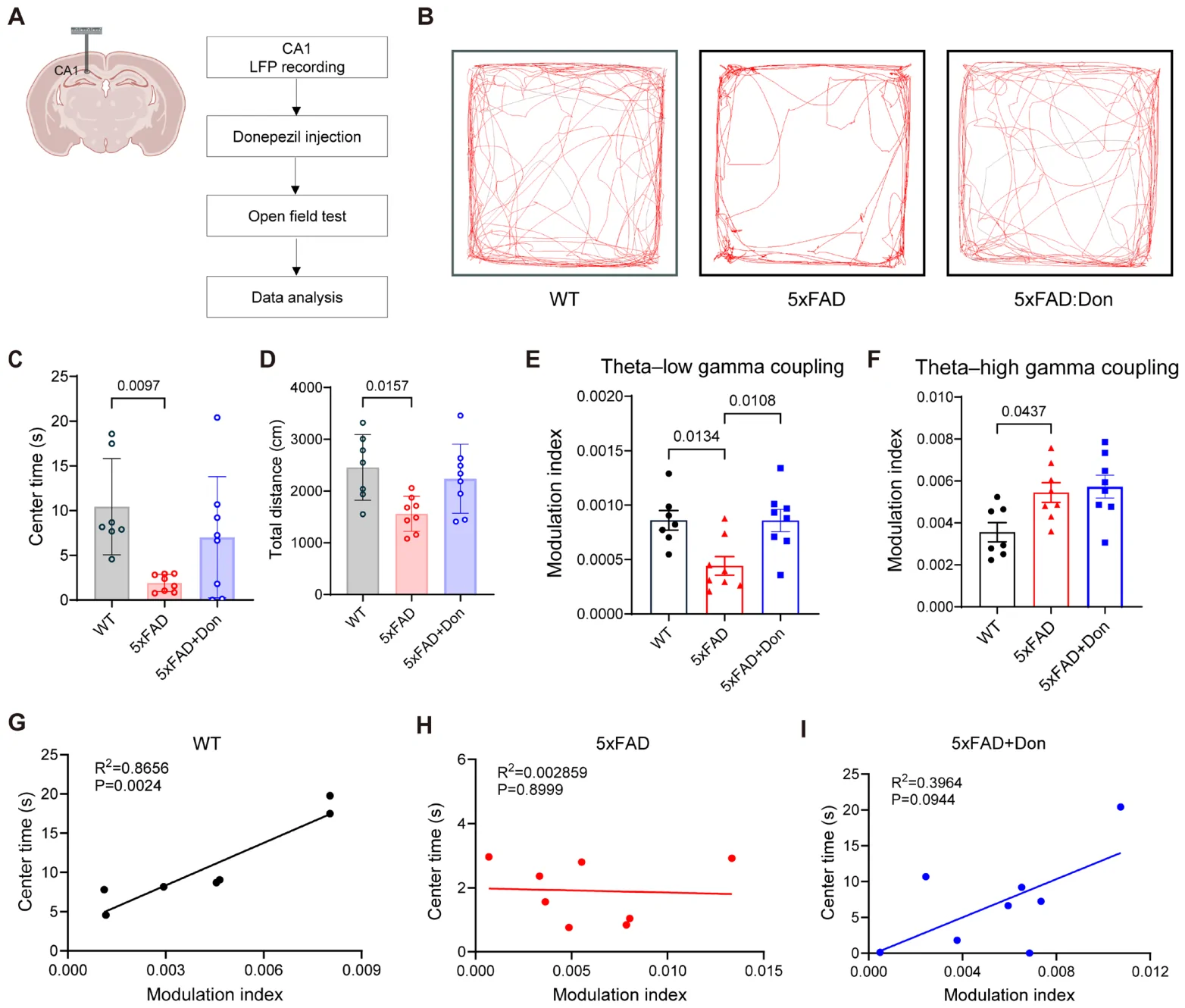
Open-field behavior and CA1 theta–gamma coupling after donepezil treatment in 5xFAD mice. (**A**) Experimental workflow for CA1 LFP recording, donepezil administration, open-field testing, and data analysis. (**B**) Representative open-field trajectories from WT, untreated 5xFAD, and donepezil-treated 5xFAD (5xFAD + Don) mice. (**C**,**D**) Time spent in the center and total distance traveled, respectively. (**E**,**F**) Theta–low-gamma and theta–high-gamma MI during open-field exploration, respectively. (**G**–**I**) Associations between theta–high-gamma MI and center time in WT, untreated 5xFAD, and 5xFAD + Don mice, respectively; lines indicate simple linear regression. Data in (**C**–**F**) are shown as the mean ± SEM. Black circles, red triangles, and blue squares denote WT, untreated 5xFAD, and 5xFAD + Don mice, respectively. Each symbol represents one mouse (animal-level value): WT, *n* = 7; 5xFAD, *n* = 8; 5xFAD + Don, *n* = 8. Group comparisons in (**C**–**F**) were performed using one-way analysis of variance followed by Tukey’s multiple-comparisons test. Exact *p* values for the indicated comparisons were: (**C**) WT versus 5xFAD, *p* = 0.0097; (**D**) WT versus 5xFAD, *p* = 0.0157; (**E**) WT versus 5xFAD, *p* = 0.0134 and 5xFAD versus 5xFAD + Don, *p* = 0.0108; and (**F**) WT versus 5xFAD, *p* = 0.0437. Linear-regression results were: (**G**) R^2^ = 0.8656, *p* = 0.0024; (**H**) R^2^ = 0.002859, *p* = 0.8999; and (**I**) R^2^ = 0.3964, *p* = 0.0944.

To examine how cholinergic modulation affects exploratory behavior and hippocampal network activity in 5xFAD mice, CA1 LFPs were recorded during the open-field test after seven consecutive days of donepezil treatment (Figure 4A). Representative movement trajectories from WT, untreated 5xFAD, and donepezil-treated 5xFAD mice are shown in Figure 4B.

Behavioral analysis showed that untreated 5xFAD mice spent less time in the center of the arena than WT mice (Figure 4C; *p* = 0.0097) and traveled a shorter total distance (Figure 4D; *p* = 0.0157). Center time and total distance were numerically higher in donepezil-treated than in untreated 5xFAD mice, but these pairwise differences were not statistically significant.

We next analyzed theta–gamma phase–amplitude coupling during open-field exploration. Theta–low-gamma coupling was lower in untreated 5xFAD mice than in WT mice (Figure 4E; *p* = 0.0134), whereas donepezil-treated 5xFAD mice showed higher theta–low-gamma coupling than untreated 5xFAD mice (Figure 4E; *p* = 0.0108). Theta–high-gamma coupling was higher in untreated 5xFAD mice than in WT mice (Figure 4F; *p* = 0.0437). Although the mean remained numerically elevated in the donepezil-treated group, no significant pairwise comparison involving that group was indicated.

Correlation analysis showed a significant positive association between the theta–high-gamma modulation index and center time in WT mice (Figure 4G; R^2^ = 0.8656, *p* = 0.0024). This relationship was absent in untreated 5xFAD mice (Figure 4H; R^2^ = 0.002859, *p* = 0.8999). In donepezil-treated 5xFAD mice, a positive but non-significant trend was observed (Figure 4I; R^2^ = 0.3964, *p* = 0.0944).

Together, these findings indicate that untreated 5xFAD mice exhibit reduced exploratory behavior and abnormal CA1 theta–gamma coupling during open-field exploration. Donepezil significantly increased theta–low-gamma coupling relative to untreated 5xFAD mice, whereas its effect on theta–high-gamma coupling was not resolved by the present pairwise comparisons.

### 3.5. Y-Maze Exploration Reveals State-Dependent Theta–Gamma Coupling Abnormalities in 5xFAD Mice

**Figure 5:**
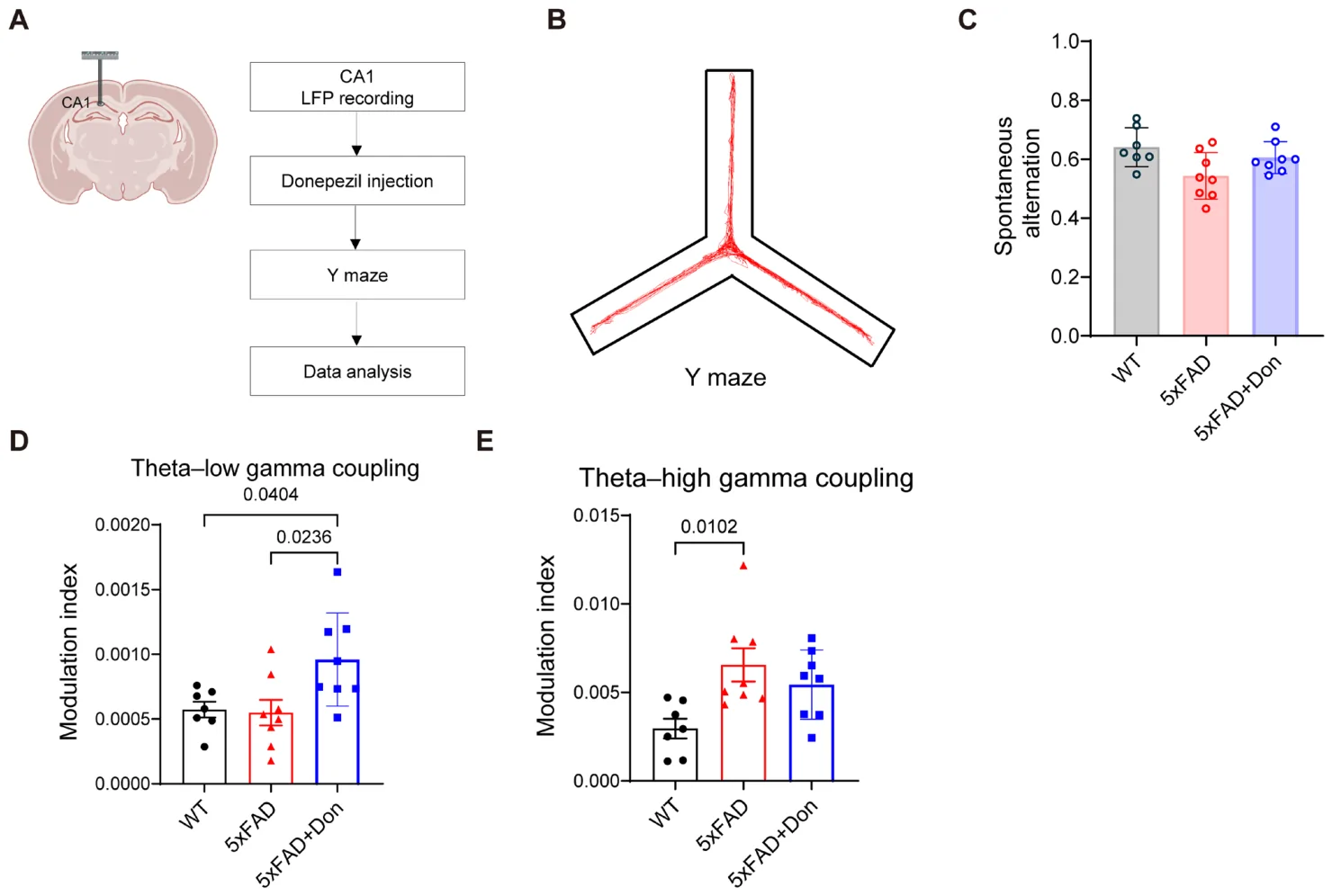
Y-maze performance and CA1 theta–gamma coupling after donepezil treatment in 5xFAD mice. (**A**) Experimental workflow for CA1 LFP recording, donepezil administration, Y-maze testing, and data analysis. (**B**) Representative trajectory during Y-maze exploration. (**C**) Spontaneous alternation in WT, untreated 5xFAD, and donepezil-treated 5xFAD (5xFAD + Don) mice. (**D**,**E**) Theta–low-gamma and theta–high-gamma MI during Y-maze exploration, respectively. Data are shown as the mean ± SEM. Black circles, red triangles, and blue squares denote WT, untreated 5xFAD, and 5xFAD + Don mice, respectively. Each symbol represents one mouse (animal-level value): WT, *n* = 7; 5xFAD, *n* = 8; 5xFAD + Don, *n* = 8. Group comparisons were performed using one-way analysis of variance followed by Tukey’s multiple-comparisons test. Exact *p* values for the indicated comparisons were: (**D**) WT versus 5xFAD + Don, *p* = 0.0404; 5xFAD versus 5xFAD + Don, *p* = 0.0236; and (**E**) WT versus 5xFAD, *p* = 0.0102.

To evaluate hippocampal network activity during spatial exploration, CA1 LFPs were recorded while WT, untreated 5xFAD, and donepezil-treated 5xFAD mice freely explored the Y-maze after seven consecutive days of treatment (Figure 5A). Representative movement trajectories during the Y-maze test are shown in Figure 5B. Behavioral analysis showed no significant difference in spontaneous alternation among the three groups (Figure 5C), suggesting that overt impairment in spontaneous alternation was not detected under these experimental conditions.

We next examined theta–gamma phase–amplitude coupling during Y-maze exploration. Theta–low-gamma coupling did not differ significantly between WT and untreated 5xFAD mice. Donepezil-treated 5xFAD mice showed higher theta–low-gamma coupling than both WT mice (Figure 5D; *p* = 0.0404) and untreated 5xFAD mice (Figure 5D; *p* = 0.0236). Theta–high-gamma coupling was higher in untreated 5xFAD mice than in WT mice (Figure 5E; *p* = 0.0102). No significant pairwise comparison involving the donepezil-treated group was indicated for theta–high-gamma coupling.

Together, these findings indicate that CA1 theta–gamma coupling is altered during Y-maze exploration despite the absence of a significant group difference in spontaneous alternation. Donepezil increased theta–low-gamma coupling relative to untreated 5xFAD mice, whereas its effect on theta–high-gamma coupling remained inconclusive.

## 4. Discussion

The present study was designed to test whether hippocampal theta–gamma coupling is altered in a frequency-specific and behavioral-state-dependent manner in 5xFAD mice and whether short-term cholinergic enhancement differentially affects low- and high-gamma-associated PAC components. At the animal level, untreated 5xFAD mice showed elevated theta–high-gamma coupling across home-cage, open-field, and Y-maze conditions, whereas theta–low-gamma coupling was reduced during home-cage activity and open-field exploration but not during Y-maze exploration. Seven days of donepezil treatment significantly increased theta–low-gamma coupling during open-field and Y-maze exploration and produced a partial numerical shift toward WT values under home-cage conditions. For theta–high-gamma coupling, however, the treated group did not differ significantly from either WT or untreated 5xFAD mice in the three-group analyses. Thus, the data support frequency- and state-dependent PAC abnormalities and exploratory treatment-associated patterns, while the effect of donepezil on theta–high-gamma coupling remains unresolved.

A major implication of the present study is that AD-related abnormalities in cross-frequency coordination cannot be adequately described as a uniform increase or decrease in theta–gamma coupling. Previous studies in AD animal models have more commonly reported reductions in theta–gamma PAC, but the affected gamma component and the conditions under which the reduction was detected have varied considerably. Goutagny et al. reported impaired hippocampal theta–gamma coupling in one-month-old TgCRND8 mice before marked Aβ overproduction, suggesting that cross-frequency disorganization can emerge at a very early stage of amyloid-related disease development [45]. These PAC findings occur within a broader literature showing both Aβ-induced gamma desynchronization and preserved gamma rhythmogenesis under different experimental conditions [46,47] while highlighting the relevance of parvalbumin-interneuron and cholinergic-sensitive theta mechanisms to network coordination [48,49]. In J20-APP mice, Etter et al. found reduced CA1 slow-gamma activity and theta–slow-gamma PAC during spatial-memory retrieval. Importantly, optogenetic stimulation of medial septal parvalbumin-positive neurons at 40 Hz, but not 80 Hz, restored slow-gamma coupling and improved memory retrieval, providing evidence that the reduction in theta–slow-gamma PAC was both frequency specific and functionally relevant [50].

Behavioral state and task phase also appear to influence the direction and magnitude of PAC abnormalities. In pre-plaque TgF344-AD rats, van den Berg et al. observed reduced hippocampal theta–gamma PAC predominantly during wake immobility, whereas the abnormality was less evident during active exploration [33]. This finding indicates that PAC deficits can be masked or modified by changes in locomotion and behavioral engagement. More recently, Ai et al. reported reduced theta–low-gamma PAC in APP/PS1 mice during the encoding phase of a spatial working-memory task, together with reduced theta- and low-gamma-directed information flow from the ventral hippocampus to the medial prefrontal cortex [34]. These animal studies generally converge with the reduction in theta–low-gamma coupling observed in the present study under home-cage and open-field conditions. However, the absence of a significant theta–low-gamma reduction during Y-maze exploration in our study differs from the encoding-related deficit reported in APP/PS1 mice. This difference may reflect variations in the disease model, hippocampal subregion, task structure, memory demand, recording epoch, or the distinction between local CA1 coupling and interregional hippocampal-prefrontal communication.

In contrast to the relatively consistent evidence for reduced theta-low or theta–slow-gamma coupling, increased theta–high-gamma PAC has received less attention in AD animal models. Many previous studies either analyzed a broad gamma band or focused primarily on slow-gamma frequencies below approximately 60 Hz, potentially obscuring opposite changes in faster gamma components. A recent wideband intracranial EEG study in young 5xFAD mice reported increased high-frequency oscillation activity and abnormal coupling between slow-wave phase and HFO amplitude before overt plaque deposition and neuroinflammatory changes [35]. Although slow-wave–HFO coupling is physiologically and analytically distinct from the theta–high-gamma PAC examined here, both findings support the possibility that excessive organization of higher-frequency activity may emerge early in amyloid-driven network dysfunction. The elevation of theta–high-gamma PAC in untreated 5xFAD mice across all three behavioral contexts extends the previous literature by showing that reduced lower-gamma coupling and enhanced higher-gamma coupling can coexist within the same hippocampal network. PAC and fast-ripple/high-frequency activity should be viewed as complementary rather than interchangeable measures: PAC quantifies coordination across frequency bands, whereas fast ripples and epileptiform events may more directly index pathological hyperexcitability.

Studies in patients with AD similarly show that theta–gamma coupling abnormalities are directionally heterogeneous. Using scalp EEG during an N-back working-memory task, Goodman et al. found that theta–gamma coupling was lowest in patients with AD, intermediate in individuals with mild cognitive impairment, and highest in cognitively healthy controls. Coupling strength was also positively associated with working-memory performance, supporting a relationship between reduced task-related theta–gamma coordination and cognitive impairment [51]. In a longitudinal resting-state EEG study, Musaeus et al. found significantly lower global gamma/theta coupling in individuals with progressive MCI than in those with stable MCI; the lower values in the broader AD and MCI groups relative to healthy controls did not reach significance [52]. Source-reconstructed MEG recordings have also demonstrated reduced theta–gamma PAC in the left parahippocampal cortex of patients with AD, with more pronounced bilateral abnormalities in patients exhibiting subclinical epileptiform activity [32].

Conversely, increased theta–gamma coupling has also been observed in human AD. Chen et al. reported increased global resting-state theta–gamma coupling in patients with AD and increased right temporal and parietal coupling in individuals with MCI compared with healthy controls [53]. The right temporal region showed an apparent progression from healthy controls to MCI and AD, suggesting that increased coupling may accompany disease-related network reorganization in some resting-state conditions. Other reports of enhanced low-frequency-gamma cross-frequency interaction in AD have used bicoherence or related nonlinear connectivity measures rather than conventional phase-amplitude coupling indices. Such findings are relevant to the broader concept of abnormal cross-frequency coordination but should not be considered directly equivalent to local PAC measured from hippocampal LFPs.

Taken together, the existing literature does not support a single direction of theta–gamma PAC alteration in AD. Reduced coupling has frequently been associated with impaired mnemonic coordination, weakened hippocampal communication, or progression from stable to clinically worsening cognitive impairment. In contrast, increased coupling may reflect compensatory recruitment, excessive synchronization, altered excitation–inhibition balance, or pathological network hyperexcitability. These interpretations are not mutually exclusive because different PAC components may be altered in opposite directions within the same network. The present animal-level results support this possibility: theta–low-gamma coupling was reduced in a behavioral-state-dependent manner, whereas theta–high-gamma coupling was elevated in untreated 5xFAD mice across the three recording contexts.

The present study also differs from most previous work in three respects. First, local CA1 PAC was examined in the same disease model across home-cage activity, open-field exploration, and Y-maze testing, enabling behavioral-state effects to be assessed under otherwise comparable recording and analytical conditions. Second, low- and high-gamma amplitude components were analyzed separately rather than being combined into a broad gamma band. Third, the pharmacological responsiveness of each PAC component was evaluated following donepezil treatment. Donepezil significantly increased theta–low-gamma coupling during open-field and Y-maze exploration, whereas the three-group comparisons did not establish a significant treatment effect on theta–high-gamma coupling. These results indicate that the two PAC components showed different treatment-associated patterns, but they do not establish a statistically confirmed differential response or support classifying theta–high-gamma coupling as treatment resistant.

Nevertheless, direct comparisons across studies should be made cautiously. Published studies differ in animal model, age, pathological stage, recording region, behavioral task, vigilance state, electrode type, reference configuration, frequency-band definition, and PAC estimator. Moreover, scalp EEG and MEG measurements in patients do not isolate local CA1 activity and may reflect interactions among multiple cortical and subcortical generators. Accordingly, the present findings should be interpreted as evidence for frequency- and state-specific PAC abnormalities in an accelerated amyloid-dominant mouse model rather than as a direct electrophysiological equivalent of PAC alterations in human sporadic AD.

The opposing changes in theta–low-gamma and theta–high-gamma PAC may be interpreted in relation to the functional organization of hippocampal gamma subbands. In CA1, low-gamma activity has often been associated with CA3–CA1 communication, whereas faster gamma activity has been linked to entorhinal cortex–CA1 input. Although this pathway-based interpretation is not absolute and may vary with behavioral state, recording location, and analytical definitions of gamma subbands, it provides a useful framework for interpreting the present findings. Reduced theta–low-gamma coupling may reflect impaired temporal coordination of CA3-driven inputs to CA1, which could affect hippocampal computations involved in memory retrieval, pattern completion, and exploratory processing. By contrast, elevated theta–high-gamma coupling may reflect abnormal entorhinal–hippocampal synchronization, compensatory recruitment of cortical input, or pathological network hyperexcitability. This interpretation is consistent with broader evidence that amyloid-related pathology can disturb excitation–inhibition balance and promote aberrant synchronization in hippocampal circuits [48,54]. However, the present CA1 LFP recordings cannot determine the anatomical or cellular source of the PAC abnormalities. Therefore, the CA3–CA1 and entorhinal cortex–CA1 explanation should be regarded as a plausible circuit-level hypothesis rather than a definitive mechanism. Elevated theta–high-gamma coupling may therefore reflect persistent network dysfunction, altered excitation–inhibition balance, interneuron dysfunction, amyloid-associated hyperexcitability or epileptiform activity, or residual signal artifact; the present data cannot assign a single mechanism.

The effects of donepezil further support the idea that theta–low-gamma and theta–high-gamma coupling represent distinct network readouts. Donepezil significantly increased theta–low-gamma coupling during open-field and Y-maze exploration and produced a nonsignificant numerical shift toward WT values under home-cage conditions. Since donepezil increases acetylcholine availability by inhibiting acetylcholinesterase, these findings suggest that theta–low-gamma coupling is sensitive to short-term cholinergic modulation in behaviorally engaged states. Septo-hippocampal cholinergic signaling is closely involved in hippocampal theta rhythm generation, attentional and arousal states, synaptic plasticity, and memory-related processing [43,49]. Enhancement of theta–low-gamma coupling may therefore indicate altered temporal coordination of hippocampal ensembles rather than a simple increase in oscillatory power. This interpretation is consistent with the absence of significant pre- versus post-dosing changes in theta- and gamma-band power.

The revised comparisons do not establish whether elevated theta–high-gamma coupling persists after donepezil treatment. Although untreated 5xFAD mice differed significantly from WT mice across the three behavioral contexts, the donepezil-treated group did not differ significantly from either WT or untreated 5xFAD mice in the corresponding three-group analyses. This intermediate statistical pattern may reflect a partial shift, biological variability, or limited power, and it should not be interpreted as proof of either treatment resistance or complete rescue. Longer treatment windows, larger cohorts, and within-subject analyses will be needed to define the responsiveness of theta–high-gamma coupling.

The behavioral findings also require cautious interpretation. Untreated 5xFAD mice showed reduced open-field distance and center exploration time, indicating altered locomotor or exploratory behavior. Both measures were numerically higher after donepezil treatment, but the pairwise differences versus untreated 5xFAD mice were not statistically significant. Y-maze spontaneous alternation did not differ significantly among groups under the present conditions. These results indicate that electrophysiological modulation and behavioral rescue were not tightly coupled. This dissociation is not necessarily unexpected because behavioral measures can be influenced by locomotor activity, anxiety-like behavior, motivation, sensory function, and testing conditions. PAC analysis may provide additional information about circuit-level drug engagement that is not captured by coarse behavioral endpoints. Nevertheless, the present correlation analyses do not establish causality. The data do not demonstrate that PAC abnormalities directly cause behavioral impairment or that donepezil-induced PAC changes mediate behavioral improvement. At four months of age, amyloid-associated network dysfunction may be detectable before a robust spontaneous-alternation deficit emerges, and the non-significant Y-maze result should not be interpreted as evidence that 5xFAD mice lack cognitive impairment. A within-group PAC–behavior association and a group-mean PAC difference test different hypotheses; therefore, a treatment-group association can change without a significant shift in mean PAC. With the present sample size, the group-specific correlations remain sensitive to restricted ranges and outliers.

More broadly, the present study supports the potential value of animal-level PAC measures as candidate functional readouts in preclinical AD research. A useful functional biomarker should be sensitive to disease-associated circuit changes, responsive to pharmacological modulation, and interpretable within a biologically plausible framework. In this study, theta–high-gamma coupling was elevated in untreated 5xFAD mice across behavioral states, whereas theta–low-gamma coupling responded to donepezil during open-field and Y-maze exploration. These complementary properties suggest that separating gamma subbands may provide more informative readouts than analyzing broadband gamma activity alone. Importantly, PAC should not yet be regarded as a validated surrogate endpoint. Rather, it should be considered a candidate circuit-level measure that may complement behavioral, histological, and molecular endpoints in preclinical AD studies.

An important limitation concerns the disease model used in this study. The 5xFAD model is an accelerated, amyloid-dominant transgenic model driven by five familial AD-associated mutations. It does not reproduce the prolonged, multifactorial disease development characteristic of sporadic late-onset AD and lacks progressive human-like tau aggregation and neurofibrillary tangle formation [36,37]. Accordingly, the PAC abnormalities observed here should primarily be interpreted as electrophysiological consequences associated with aggressive Aβ-related pathology rather than as a complete representation of network dysfunction across the full spectrum of human AD. In addition, neuronal overexpression of mutant APP and PSEN1 may produce effects that are not entirely attributable to endogenous Aβ pathology. Future studies should therefore determine whether the frequency- and state-dependent PAC patterns identified here are reproduced in aging-based or knock-in models, models incorporating sporadic AD risk factors, and models exhibiting combined amyloid and tau pathology.

Additional limitations should also be acknowledged. First, recordings were restricted to hippocampal CA1, which limits the ability to determine whether the observed PAC abnormalities originate from local CA1 microcircuits, CA3 input, entorhinal input, medial septal modulation, or broader hippocampal network interactions. Future studies should include simultaneous recordings from CA3, CA1, dentate gyrus, entorhinal cortex, and medial septum. Second, only short-term donepezil treatment was examined. Longer treatment paradigms are needed to determine whether sustained cholinergic enhancement can modify theta–high-gamma coupling or improve behavioral performance. Third, electrophysiological measures were not directly correlated with amyloid burden, synaptic markers, interneuron function, or cholinergic signaling markers. Combining LFP recordings with histological and molecular analyses will be important for establishing mechanistic links between pathology and PAC abnormalities. Fourth, although the present analyses used each animal as an independent biological replicate, the small cohort sizes limit power for detecting treatment-associated effects; larger cohorts and independent replication are therefore needed. Finally, causal experiments using optogenetic, chemogenetic, or closed-loop stimulation approaches will be needed to determine whether restoring specific PAC components can modify cognitive outcomes.

Several design limitations constrain treatment- and state-specific interpretation. Because no time-matched vehicle-treated 5xFAD group was included, pre-/post-treatment differences cannot be attributed exclusively to donepezil; repeated testing, habituation, handling, injection stress, and elapsed time may also have contributed. The absence of a WT-plus-donepezil group also prevents determination of whether the observed changes are disease-specific or reflect a general pharmacological effect. We therefore use the term treatment-associated change rather than normalization. In addition, home-cage data were not stratified by sleep/wake state, and task data were not matched for movement speed, immobility, or exploration. Differences in behavioral-state composition may therefore have contributed to the PAC findings. Only male mice were studied, further limiting generalizability across sex.

Analytical limitations also require emphasis. No phase-shuffling, permutation, or surrogate threshold was applied, and no dedicated epileptiform-event analysis was performed. Residual coupling caused by non-sinusoidal waveform shape, broadband transients, sharp-wave or epileptiform activity, movement, or filtering therefore cannot be excluded, particularly for high-gamma PAC. The modest band-boundary sensitivity check is supportive but not independent validation. Finally, cholinergic target engagement was not measured directly, and the lack of matched pathological measurements prevents attribution of PAC changes to amyloid burden, synaptic loss, interneuron dysfunction, cholinergic signaling, or neuroinflammation.

## 5. Conclusions

This study shows that hippocampal CA1 theta–gamma PAC is altered in a frequency-specific and behavioral-state-dependent manner in 5xFAD mice. Untreated 5xFAD mice showed reduced theta–low-gamma coupling in home-cage and open-field conditions and elevated theta–high-gamma coupling across all three behavioral contexts. Donepezil increased theta–low-gamma coupling during open-field and Y-maze exploration, whereas its effect on theta–high-gamma coupling was not resolved by the present comparisons. These findings demonstrate that low- and high-gamma-associated PAC components can be dissociated across behavioral states and pharmacological modulation. Animal-level PAC analysis may provide a complementary approach for evaluating circuit dysfunction and treatment responsiveness in preclinical AD models, but longitudinal, mechanistic, and pathology-linked validation is required before PAC can be considered a reliable biomarker of disease progression or therapeutic efficacy.

## Data Availability

The original contributions presented in this study are included in the article and Supplementary Materials. Further inquiries can be directed to the corresponding author.

## Supplementary Materials

The following supporting information can be downloaded at: https://www.mdpi.com/article/10.3390/brainsci16080816/s1, Table S1. Primer sequences used for 5xFAD mouse genotyping.

## Abbreviations

The following abbreviations are used in this manuscript:

AD	Alzheimer’s disease
LFP	local field potential
Don	donepezil
OFT	open-field test
PAC	phase-amplitude coupling
MI	modulation index
WT	wild type
PSD	power spectral density
