# Supplementary material for: State-Dependent Alterations of Hippocampal Theta–Gamma Coupling in 5xFAD Mice and Their Differential Modulation by Donepezil
Source: Brain Sci. 2026 Jul 31;16(8):816. doi: 10.3390/brainsci16080816 (PMC13511096; doi:10.3390/brainsci16080816)
Supplement: Supplementary file 1 [file brainsci-16-00816-s001.zip › brainsci-4407478-supplementary.pdf]

Supplementary Materials

State-Dependent Alterations of Hippocampal Theta-Gamma Coupling in 5xFAD Mice and Their Differential Modulation by Donepezil

Haodong Wang, Yajun Wang, Zhengyang Lv, Yueting Deng, Chao Pan, Yuping Li, and Zikai Zhou

Table S1. Primer sequences used for 5xFAD mouse genotyping.

Primer	Sequence (5′→3′)	Primer Type
**P1**	CGGGCCTCTTCGCTATTAC	Mutant Reverse
**P2**	ACCCCCATGTCAGAGTTCCT	Common
**P3**	TATACAACCTTGGGGGATGG	Wild type Reverse
